# Acquired angioedema

**DOI:** 10.1186/1710-1492-6-14

**Published:** 2010-07-28

**Authors:** Marco Cicardi, Andrea Zanichelli

**Affiliations:** 1 Dipartimento di Scienze Cliniche "Luigi Sacco" Università di Milano, Milano Italy, Ospedale L.Sacco Milano, Italy

## Abstract

Acquired angioedema (AAE) is characterized by acquired deficiency of C1 inhibitor (C1-INH), hyperactivation of the classical pathway of human complement and angioedema symptoms mediated by bradykinin released by inappropriate activation of the contact-kinin system. Angioedema recurs at unpredictable intervals, lasts from two to five days and presents with edema of the skin (face, limbs, genitals), severe abdominal pain with edema of the gastrointestinal mucosa, life-threateing edema of the upper respiratory tract and edema of the oral mucosa and of the tongue. AAE recurs in association with various conditions and particularly with different forms of lymphoproliferative disorders. Neutralizing autoantibodies to C1-INH are present in the majority of patients. The therapeutic approach to a patient with AAE should first be aimed to avoid fatalities due to angioedema and then to avoid the disability caused be angioedema recurrences. Acute attacks can be treated with plasma-derived C1-INH, but some patients become non-responsive and in these patients the kallikrein inhibitor ecallantide and the bradykinin receptor antagonist icatibant can be effective. Angioedema prophylaxis is performed using antifibrinolytic agents and attenuated androgens with antifibrinolytic agents providing somewhat better results. Treatment of the associated disease can resolve AAE in some patients.

## Review

### The symptoms

The three key elements of the syndrome commonly referred to as acquired angioedema (AAE), which was first described by Caldwell in 1972 [1], are acquired deficiency of C1 inhibitor (C1-INH), hyperactivation of the classical pathway of human complement and recurrent angioedema symptoms. It is considered a very rare condition with just more than 100 patients reported in the literature [2]. In absence of epidemiological data, we can only speculate about its prevalence. In our list of angioedema patients, we found 1 AAE every 10 patients with the hereditary form of C1-INH deficiency (hereditary angioedema, HAE). HAE minimal prevalence in the population is 1.41/100,000 and usual estimated prevalence between 1:10,000 and 1:50,000 [3,4]. Therefore, a very crude estimated prevalence of AAE could range between 1:100,000 and 1:500,000. We believe that the actual number is much higher than this because the condition is frequently unrecognized.

**Figure 1:**
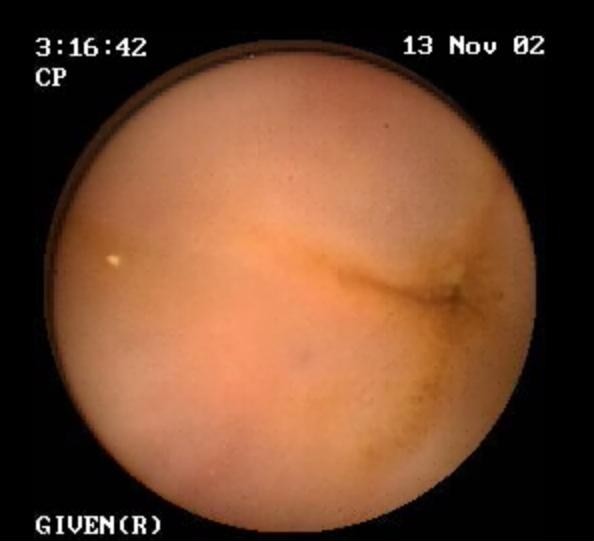
**Capsule endoscopy showing bowel occlusion due to angioedema of the gastrointestinal mucosa in a patient with C1-INH deficiency suffering from acute abdominal pain**.

**Figure 2:**
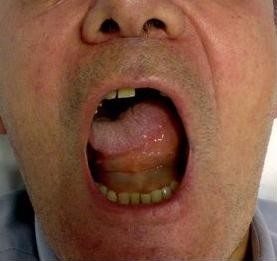
**Edema of the tongue in a patient with acquired angioedema**.

From the clinical point of view the angioedema symptoms that characterize AAE can not be differentiated from those present in HAE patients who have a deficiency of C1-INH due to mutations in one of the two alleles coding for this protein [5]. This could be anticipated based on the fact that in both forms angioedema is mediated by bradykinin episodically released by inappropriate activation of the contact-kinin system lacking its major physiologic regulator C1-INH [6,7]. Thus, similar to HAE patients, patients with AAE have no major urticaria flare. Angioedema recurs at unpredictable intervals, lasting from two to five days and presenting with disfiguring, non pitting, non-pruritic edema of the skin (face, limbs, genitals), severe abdominal pain for edema of the gastrointestinal mucosa leading to temporary bowel occlusion (Figure 1) [8], life-threateing edema of the upper respiratory tract and edema of the oral mucosa and of the tongue [2]. The only significant clinical difference between HAE and AAE is the age of onset of symptoms (Table 1): within the second decade of life for more than 90% of patients with HAE, after the fourth decade for those with AAE. Some additional minor differences can be found looking at different rates of recurrences at specific sites. Angioedema of the gastrointestinal mucosa causing abdominal pain is reported by nearly 80% of patients with HAE while less than 50% of our AAE patients and around 30% of those from Bouillet et al [9] reported such symptoms. Nevertheless, presentation of AAE with abdominal symptoms has been reported in our series and in the literature [10]. Cutaneous angioedema in HAE patients is typically localized to the extremities. Even if this location is also present in patients with AAE, in them angioedema recurs more frequently in the face than in the limbs [9] and we also noticed a rather frequent involvement of tongue and uvula; (Figure 2).

**Table 1:** Differences between acquired and hereditary angioedema due to C1-INH deficiency.

	**Onset < 20 y.o**.	**Onset >40 y.o**.	Abdominal	C1q < 50%
	**% pts**.	**% pts**.	**% pts**.	%pts
Acquired angioedema	0	94	48	70
Hereditary angioedema	12	3	87	< 5

Data are based on a personal case list of 43 patients with acquired and 448 with hereditary angioedema.

### The pathogenesis of the acquired defect of C1 inhibitor and the associated disease

In the first patients reported by Caldwell [1], AAE occurred in presence of lymphoma. This association has been repeatedly confirmed in subsequent patients [11-13] and even if lymphoma is not the only disease associated with AAE, it remains the preeminent disease association. Along with lymphoma, other benign forms of lymphoproliferation, namely monoclonal gammopathy of uncertain significance (MGUS), have been reported with high frequency in association with AAE. Capacity of lymphoma cells to deplete C1-INH or to cause its consumption through C1 activation, and the possibility to treat AAE by curing the underlying lymphoma, linked the lymphoproliferative disease to the pathogenesis of AAE [1,11-15]. In 1985, neutralizing autoantibodies to C1-INH were described in a few otherwise healthy patients [16,17]. Anti-C1-INH autoantibodies recognize epitopes around the reactive center of C1-INH and by binding these epitopes render the protein functionally inactive and/or increase its catabolism [18,19]. Autoantibody-mediated AAE seemed to be, at the beginning, a new type of AAE in which autoreactive immunoglobulins, instead of lymphoma tissues, was the cause of C1-INH depletion [20]. Re-evaluation of existing and new AAE patients demonstrated that autoantibodies could be present along with lymphoma and that the M component detected in several AAE patients corresponded to the anti-C1-INH autoantibodies [21]. Based on these findings, we can now see AAE as a condition with different form of abnormal B cell proliferation progressing from autoreactivity to malignant lymphoma. Whether the different degrees of lymphoproliferation found in AAE patients are evolutionary stages of the same process starting from expansion of anti-C1-INH autoreactive clone(s) has not yet been clarified [22].

Although lymphoproliferative diseases represent the main group encountered in AAE and a direct pathogenetic relationship between the two conditions can not be questioned, SLE, different neoplasias and infections have also been described in association with AAE [23-33]. The possibility chance association for some of these conditions can not be completely ruled out.

### The diagnosis

AAE is first suspected in patients aged 40 or above who present with recurrent cutaneous and/or mucosal angioedema without urticaria, without an evident triggering factor, and without family history of angioedema. Measurement of C1-INH and C4 antigen in such patients is the first step. If both are normal, the deficiency of C1-INH is very unlike. If both are low (with C1-INH below 50% of normal on two separate determinations) diagnosis of C1-INH deficiency is made. If just C4 is reduced, C1-INH functional activity needs to be determined and if low (below 50% of normal on two separate determinations) diagnosis of C1-INH deficiency is made. Once this diagnosis has been made, additional testing is necessary to distinguish between inherited and acquired deficiency. This testing includes determination of C1q which is reduced in 70% of patients with AAE and is normal in HAE. If C1q is reduced, diagnosis of AAE can be established. If C1q is normal, autoantibodies to C1-INH can be investigated and their presence at high titre allows diagnosing AAE. If antibodies are negative, the diagnosis of AAE is assumed when complete screening of C1-INH gene gives no evidence of mutations affecting C1-INH protein. Major limits to this procedure are the inadequate availability and standardization of C1-INH functional measurements [34] and the possibility to look for anti-C1-INH autoantibodies only in a few specialized research laboratories. Therefore, careful harmonization of clinical and laboratory findings is mandatory to establish the diagnosis of AAE.

Patients with this diagnosis should undergo basic testing for lymphoproliferative and autoimmune disease. In addition to complete physical exam, we suggest that all patients have laboratory testing for complete blood cell count with differential, serum protein electrophoresis, antinuclear antibodies, chest X ray and abdominal ultrasound assessing lymphoid tissue. Due to the limited recurrences of other associated diseases, we do not see the need to systematically screen for other neoplasia or infection without specific clinical indications.

### The treatment

The therapeutic approach to a patient with AAE should first be aimed to avoid fatalities due to angioedema and then to avoid the disability caused be angioedema recurrences. Angioedema- related fatalities derive from laryngeal edema. Based on the efficacy of replacement therapy with plasma-derived C1-INH in reverting laryngeal edema in patients with HAE [35], the same approach has been used for AAE. This treatment works in the majority but not in all AAE patients and in our experience some patient with AAE become progressively non responsive to plasma-derived C1-INH or need increased doses [5]. No other treatment for angioedema attacks has been extensively used in patients with AAE and therefore there is no established therapeutic alternative to plasma- derived C1-INH for life-threatening attacks. Non-responsive patients have just been assisted with invasive procedures aimed to maintain patency of upper airways during emergency. In recent years in a few AAE patients we have used two of the new treatments that have been proposed for HAE acute attacks: the kallikrein inhibitor ecallantide and the bradykinin B2 antagonist icatibant [36]. Since refractoriness to plasma-derived C1-INH is due to its autoantibody-mediated rapid catabolism, the use of drugs different from C1-INH but active in reversing HAE attacks have very good rationale for being effective in AAE. In fact from our limited experience response is extremely favourable. We treated 2 facial attacks in two patients with ecallantide and 1 laryngeal and 3 facial attacks in another patient with icatibant. Two of these patients were completely, and one partially, non-responsive to plasma derived C1-INH. All treated attacks responded very rapidly either to ecallantide or icatibant. The critical condition of C1-INH non-responder patients and the absence of licensed drugs strongly indicate the need for off-label treatments. Therefore, we recommend all our AAE patients always have 3000 U of plasma-derived C1-INH immediately available and treat attacks with 1500 U and repeating if ineffective. In the event of laryngeal edema, resuscitation facilities should be available. For those patients who have slow or no response, ecallantide or icatibant should be provided.

Reducing disability related to angioedema recurrences can be obtained by shortening attacks with an on-demand treatment with plasma-derived C1-INH, by preventing attacks with long term prevention with antifibrinolytics or androgens, or by curing the associated disease. The latter is the first choice when the associated disease has *per se *an indication to be treated. Resolution of the associated disease results in variable degrees of resolution of AAE from symptomatic improvement to complete biochemical and clinical recovery [13-15]. Treatment of the associated disease aimed only to control angioedema symptoms requires careful risk/benefit evaluation. Since most of the time the associated disease is lymphoproliferative, the choice to start a patient on chemotherapy or immunosuppressant is not always straightforward. Long-term treatment to prevent angioedema symptoms is often used in HAE and has also been used in AAE. While in HAE androgen derivatives are very effective prophylactic agents, results may not be as good in AAE. The reason for this is not totally clear. We know that attenuated androgens can increase the plasma levels of C1-INH [37]. Even if effective androgen doses in HAE do not require a measurable increase of C1-INH in plasma, it is still possible that these drugs relay on C1-INH production and their efficacy is less when C1-INH catabolism is very rapid [38]. In contrast, antifibrinolytic agents, the other class of drugs used for symptom prophylaxis in HAE [39,40], seem to have better efficacy in AAE than in HAE. It is assumed that the effect of these drugs in C1-INH deficient patients works through their anti-plasmin activity. Plasmin is critical for angioedema symptoms in C1-INH deficiency although the role is not clearly defined [41]. In AAE patients, the instability of the systems controlled by C1-INH is higher than in HAE and active plasmin is also generated separate from angioedema symptoms reinforcing the rationale for efficacy of antifibrinolytics in this condition [42-45]. At present, we consider antifibrinolytic agents as the first choice drug for angioedema prophylaxis in AAE. Prevention of attacks with continuous infusions of plasma-derived C1-INH has been attempted with controversial results and in our opinion having very little rationale [46,47]. The half life of plasma- derived C1-INH in HAE indicates two infusions per week as the minimum to maintain protective prophylactic plasma levels [48]. Even if one assumes that AAE patients will not require a more intensive program because of the faster C1-INH catabolism and will not increase the risk of becoming resistant to plasma-derived C1-INH, this prophylactic infusion regimen seems justified only for those patients with two or more severe attacks per week, a condition that we did not find in any of our 42 AAE patients. We reserve plasma-derived C1-INH infusions for on-demand treatment of severe angioedema events and do not use this for prophylaxis.

## Competing interests

MC has consultancy agreement with Dyax, Pharming and Shire, is in advisory board and invited speaker of Dyax, Shire. CSL Behring. AZ has been Invited speaker for CSL Behring and Shire

## Authors' contributions

MC wrote the review and AZ gave critical revision and collected the personal data that are reported. Both authors have read and approved the final manuscript.
